# A Shorter-Bout of HIIT Is More Effective to Promote Serum BDNF and VEGF-A Levels and Improve Cognitive Function in Healthy Young Men

**DOI:** 10.3389/fphys.2022.898603

**Published:** 2022-06-29

**Authors:** Qing Li, Li Zhang, Zhengguo Zhang, Yuhan Wang, Chongwen Zuo, Shumin Bo

**Affiliations:** ^1^ College of Kinesiology and Health, Capital University of Physical Education and Sports, Beijing, China; ^2^ Department of Pharmacology, College of Medicine, University of Tennessee Health Science Center, Memphis, TN, United States

**Keywords:** high-intensity interval training, BDNF, VEGF-A, cognitive function, young men

## Abstract

**Objective:** The aim of this study was to investigate the effects of single bouts of high-intensity interval training (HIIT) with different duration on serum brain-derived neurotrophic factor (BDNF) and vascular endothelial growth factor-A (VEGF-A) levels and cognitive function in healthy young men.

**Methods:** Twelve healthy young men were participated in two HIIT treatments (20 min HIIT and 30 min HIIT) in a random order. BDNF, VEGF-A, cortisol, testosterone, blood lactic acid were measured and cognitive function was assessed by Stroop test (CWST) and Digital Span test (DST) before, immediately after, and 30 min after HIIT.

**Results:** 20 and 30 min HIIT increased BLa (both *p <* 0.01), cortisol (20 min HIIT: *p* < 0.05; 30 min HIIT: *p* < 0.01), and testosterone (both *p <* 0.05) levels immediately when compared with their baselines. While BLa and cortisol were significantly higher in 30 min HIIT group than in 20 min HIIT group. Moreover, BDNF concentration (*p* < 0.01), DST-F (*p* < 0.01) and DST-B (*p* < 0.05) were increased and response time of Stroop was decreased immediately after HIIT only in 20 min HIIT group. VEGF-A concentration was increased immediately after HIIT in both groups (*p* < 0.01), but after 30 min recovery, it was returned to the baseline in the 20 min HIIT group and was lower than the baseline in 30 min HIIT group (*p* < 0.05).

**Conclusion:** Twenty minutes HIIT is more effective than 30 minutes HIIT for promoting serum levels of BDNF and VEGF-A as well as cognitive function in healthy young men.

## Introduction

A growing body of evidence indicates that physical activity promotes brain health including cognitive function (Leahy et al., 2020; Ai et al., 2021). In addition, recent findings have bolstered that physical fitness was positively correlated with brain health and a reduction in risk and progression rate of a number of neurological diseases (Weaver et al., 2021). It is widely accepted that lack of time is the most common barrier for people to persistent in regular exercise, especially for young people (Fisher et al., 2015). In recent years, high-intensity interval training (HIIT), characterized by repeated bouts of high-intensity exercise interspersed by passive recovery or low-intensity exercise, has attracted growing attention as a time-efficient manner (MacInnis and Gibala, 2017; Li et al., 2021). It is recognized to improve cardiopulmonary fitness, vascular function, skeletal muscle metabolism and other metabolic processes in young people (Karlsen et al., 2017; Grace et al., 2018; Su et al., 2019; Chin et al., 2020). Nevertheless, only a few studies have investigated the brain adaptations induced by HIIT in young adults. According to these studies, we know the effects of HIIT on brain health in young people are mainly focused on the expression of neurotrophins and cognitive function (Jiménez-Maldonado et al., 2018; Oberste et al., 2018; García-Suárez et al., 2020; Inoue et al., 2020).

Brain-derived neurotrophic factor (BDNF), abundantly expressed in the nervous system, is one of the proteins that contribute to the neuron survival and growth, maintenance of synaptic connections between neurons, and improvement of brain function (Figurov et al., 1996; Li et al., 2008). Vascular endothelial growth factor-A (VEGF-A), a signaling protein stimulating angiogenesis, plays a crucial role in cognitive function by improving neural regeneration (Morland et al., 2017). Current studies indicate

that HIIT is more beneficial than moderate-intensity continuous training (MICT) on elevating serum BDNF and VEGF-A levels in healthy young men (Ferris et al., 2007; Saucedo Marquez et al., 2015; Weaver et al., 2021). Ferris et al. (2007) reports that a bout of graded exercise test (GXT) of VO_2max_ training induced higher serum BDNF level in healthy youth than one bout of 20% below the ventilatory threshold (VTh-20) training or 10% above the VTh (VTh+10) training. Similarly, Saucedo Marquez et al. (2015) show that a bout of HIIT (1-min 90%W_max_ interspersed 1-min passive rest) trigger higher serum BDNF level in healthy youth than a bout of 70%W_max_ MICT. Furthermore, Weaver et al. (2021) indicate that a bout of 4 × 30s 200%W_max_ sprint interval training (SIT) cause higher serum BDNF and VEGF-A levels in healthy youth than a bout of 65%VO_2peak_ MICT.

Cognitive function including attention, executive function, visuospatial skills and memory has been considered as one of the most essential higher functions of the human brain (Aridi et al., 2017). Several studies indicate that the effect of acute HIIT on cognitive function in healthy young adults is related to the intensity of HIIT (Hashimoto et al., 2018; Ludyga et al., 2019; Schwarck et al., 2019; Tian et al., 2021). Ludyga et al. (2019) suggests that a bout of 20 min HIIT (30s training, 30s recovery, recovery ratio = 1:1) results in significant improved inhibitory control in adolescents compared with a bout of 20 min HIIT (60s training, 30s recovery, recovery ratio = 2:1). Whereas, Schwarck et al. (2019) demonstrates that a single bout of 25 min HIIT protocol at 90% of VO_2max_ for 2 min alternating with 3 min of rest (2:3) has no impact on cognitive function in healthy males. But Hashimoto et al. (2018) observes a significant improvement of executive functions in healthy males after a bout of 28 min HIIT (4 × 4 min training at 80–90% VO_2max_ with 3 min active recovery at 50–60% VO_2max_). Furthermore, Tian et al. (2021) demonstrates that 20 min HIIT (10 × 1 min training at 85–90% HR_max_ with 1 min self-paced walking) protocol significantly improves inhibitory control of healthy youth immediately compared with 20 min MICT and resting, and the improved inhibitory control elicited by HIIT can be sustained for 90 min after exercise.

Improving healthy and avoiding lifestyle-related diseases is the aim of most people to take part in physical exercise. However, it does not mean that the prolonged duration HIIT brings people more benefits. On the contrary, the longer-duration HIIT may be ineffective or even harmful. Indeed, Farias-Junior et al. (2019) shows that prolonged-intense exercise is neurotoxic and leads to damage on cognitive function. Generally, a prescription of acute exercise involves modality, intensity, and duration. Despite relatively well described dose–response relations between exercise intensity and cognitive function, the effect of exercise duration has not yet been well examined (Chang and Etnier, 2009). A systematic review indicates that positive effects of acute HIIT on executive function are observed after exercise with total time between 11 and 20 min or between 21 and 30 min, but those with total time of less than 10 min or more than 30 min did not considerably have positive effects on executive function (Ai et al., 2021). So, regarding to a single HIIT with total time of 20 and 30 min, it is unclear which is more beneficial for promoting brain health.

As such, the aim of the present study was to compare the effects of a HIIT protocol with different durations (20 and 30 min) on serum BDNF and VEGF-A levels as well as cognitive function in healthy young men. Our results will be beneficial to provide scientific exercise guidance for young adults aimed to promote health including cognitive performance. We hypothesize that 20 min HIIT would elicit more improvement in cognitive function and serum neurotrophin level than 30 min HIIT.

## Methods

### Study Design and Participants

Twelve active men were recruited from a public university in Beijing with the following inclusion criteria: 1) 18–25 years of age; 2) BMI between 18.5 and 25 kgm^−2^; 3) no any acute and chronic diseases; 4) no surgical history within 3 months prior to the study; 5) no smoking and alcohol history; 6) right hand dominant. All participants were informed about the purpose and risks of the study and signed the informed consent. Before conducting the experiments, a minimal sample size of 12 was determined by G*Power (version 3.1, Germany) using an *α* = 0.05, a power1-β = 0.80, and an effect size = 0.40. We believe this sample size is feasible and realistic based on previous studies (Saucedo Marquez et al., 2015; Cabral-Santos et al., 2016).

**FIGURE 1:**
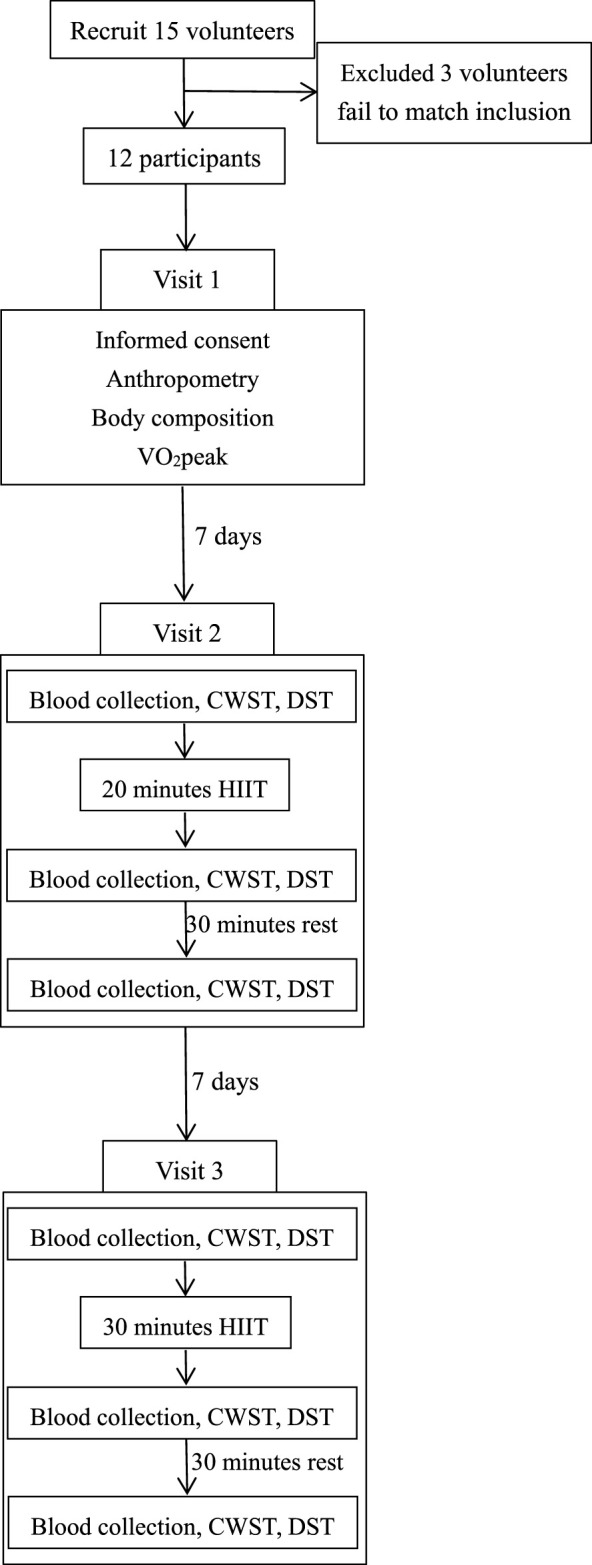
Research study design.

All the participants were required to visit our laboratory three times with 1 week interval between each visit. During the first visit, anthropometric and body composition variables were measured by bioimpedance with an Inbody™ model 770 analyzer, and VO_2peak_ was measured by gas collection system (Moxus modular oxygen uptake system, AEI technologies, USA). During the second and third visit, they were assigned to perform a HIIT protocol for 20 and 30 min, respectively. Participants were instructed to avoid caffeine and alcohol within 48 h and avoid ergogenic aids 10–12 h before every visit. During every HIIT session, participants were required to wear Polar (RS400, Finland) to record heart rate. All experimental procedures were performed between 7:00 a.m. and 12:00 p.m. A profile of the trial is shown in Figure 1. This research plan was approved by the Ethics Committee of Capital University of Physical Education and Sports (2021A32. Registered 1 September 2021). Baseline characteristics of the participants are reported in Table 1.

**TABLE 1:** Basic characteristics of the participants (M ± SD n = 12).

Variables	M±SD
Age (year)	24.33 ± 1.65
Height (m)	1.77 ± 0.06
Weight (kg)	72.38 ± 6.03
BMI (kg/m^2^)	22.18 ± 1.34
VO_2_peak (ml/kg*min)	45.33 ± 5.62
Body fat mass (%)	16.41 ± 3.58

### VO_2peak_ Measurement

During the first visit, all participants performed a graded incremental exercise test (GXT) on a cycle ergometer (ergoline100K, Germany) to determine peak oxygen uptake (VO_2peak_). Briefly, participants warmed up for 5 min by pedaling at 1 W/kg body mass. After that, the workload was increased by 25 W every minute until voluntary exhaustion (the cadence was set at 60 rpm). During the GXT, breath-by-breath pulmonary gas-exchange data were collected by AEI moxus. Heart rate (HR) was measured continuously by Polar RS400, and the rating of perceived exertion (RPE) was assessed verbally by which participants were asked to rate their perceived exertion ranging from 6 (no exertion at all) to 20 (maximal exertion). VO_2peak_ was determined when at least three of the following criteria were satisfied: 1) the respiratory exchange ratio (RER) exceeded 1.05; 2) a plateau in the VO_2_ despite increasing workload; 3) achievement of 90% of age-predicted peak HR (220-age); 4) an RPE of 19 or 20 (Suwabe et al., 2017).

### High Intensity Interval Training

During the second and third visit, the participants completed two HIIT protocols (20 min HIIT and 30 min HIIT) on a cycle ergometer (the cadence was set at 60 rpm), separately. Each HIIT session was started with a 5 min warm-up at 50 W followed by repetitive cycles composed of a 1 min bout at 85% VO_2peak_ (high-load) and 1 min active recovery at 25% VO_2peak_ (low-load) and finally finished with a 5 min cooldown at 50 W. Participants performed 15cycles in 30 min HIIT group and 10cycles in 20 min HIIT group.

### Cognitive Function

Cognitive function was assessed at baseline (pre-HIIT), immediately after training (post-HIIT) and 30 min after training (30 min post-HIIT) using color-word matching Stroop test (CWST) and Digital Span Test (DST).

**FIGURE 2:**
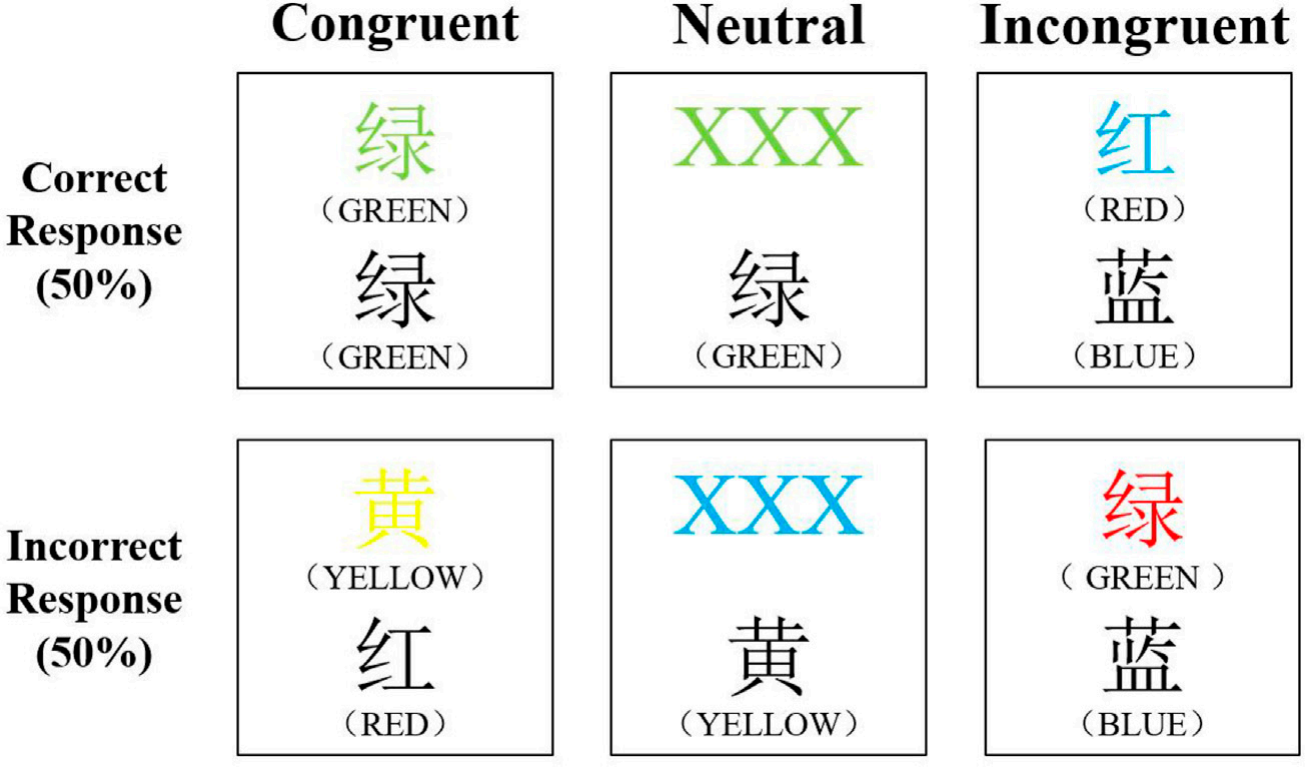
The color-word matching Stroop task.

The CWST consisted of 60 trials, including 20 congruent tests, 20 neutral tests and 20 incongruent tests, presented in a random order (Figure 2). For congruent tasks, the upper row contained the words ‘RED’, ‘YELLOW’, ‘BLUE’, or ‘GREEN’ printed in the congruent color (e.g., GREEN was printed in green) and the lower row contained color words printed in black. For neutral tasks, the upper row consisted of ‘XXX’ printed in red, yellow, blue, or green and the lower row contained the words ‘RED’, ‘YELLOW’, ‘BLUE’, or ‘GREEN’ printed in black. For incongruent tasks, the color words in the upper row were printed in an incongruent color (e.g., RED was printed in blue) and the lower row contained color words printed in black. In each trail, the target marker (+) was first presented in the center of the screen, followed by the stimulus, the stimulus remained on the screen until the response was given (Kujach et al., 2018). We instructed participants to decide whether the color of the upper word was consistent to the color name of the lower word by choosing the correct key on the keypad (F key or J key representing yes or no). The correct-answer ratio assigned to yes and no was 50%. After pressing the key, the computer recorded the participant’s response accuracy (RA) and response time (RT). All words were written in Chinese and all participants underwent three practice sessions prior to the experiment.

The DST from the Wechsler Adult Intelligence Scale (WAIS) consisted forward DST (DST-F) and backward DST (DST-B) in which the participants were requested to correctly repeat a series of increasingly length of random number sequences presented to them in the same order and reversed order, respectively. Both DST-F and DST-B were started with a four-number test at a rate of one digit per second. Correct repetition in the correct order allowed the participant to take a five-number test and so on. The task was stopped when the participant failed to repeat the last sequence correctly or recall at least two strings of the same length and score was considered to be the previous sequence length (Lavner and Rabinowitz, 2015).

### Blood Sample Collection and Analysis

The whole blood (15 ml) was drawn from an antecubital vein and the capillary blood was drawn from fingertip by a skilled nurse at pre-HIIT, post-HIIT, and 30 min post-HIIT. Blood lactate (BLa) level was measured immediately after fingertip blood collection using a digital portable lactate analyzer (SYL115Lacate-Scout, Germany). The whole blood samples were allowed to clot at room temperature for 30 min, then centrifuged at 3,000 rpm for 10 min at 4°C. The separated serum samples were frozen and kept at −80°C. The serum levels of VEGF-A, BDNF, testosterone and cortisol were measured using the following ELISA kits: human BDNF (ml900214, mlbio, China), human VEGF-A (ml060752, mlbio, China), human testosterone (ml064301, mlbio, China), human cortisol (ml711149, mlbio, China). The intra-assay coefficient of variation for the kits was <10%. All analyses were performed on an automatic microplate reader (E 601, Germany).

### Statistical Analyses

The statistical analyses were performed using SPSS26.0. All the results are presented as mean ± standard deviation (SD). The normality of data distribution was verified using the Shapiro-Wilk test. Statistical significance was set at *p* ≤ 0.05. Differences of baseline variables between two groups were conducted by independent t-tests. Factorial 2 × 3 (groups: 20 min HIIT, 30 min HIIT × time points: pre-HIIT, post-HIIT, 30 min post-HIIT) repeated measures ANOVA were used to compare the concentration of BLa, serum levels of VEGF-A, BDNF, cortisol, and testosterone as well as RT and RA between two groups at the three time points chosen. Significant main effects and interactions were assessed using the Tukey post-hoc test. η^2^ values will be reported as effect size, η^2^ was considered small if η^2^ < 0.04, and large if η^2^ > 0.36. Linear regression analysis was used to determine the relationship between changes from baseline variables. Figures were plotted by the GraphPad Prism8.0.

## Results

### BLa Responses to HIIT

As shown in Table 2, significant group by time of measurement interaction (F = 18.258, *p* < 0.001, η^2^ = 0.785), time (F = 32.080, *p* < 0.001, η^2^ = 0.865) and group (F = 39.997, *p* < 0.001, η^2^ = 0.784) main effects were found in BLa analysis. The level of BLa was significantly increased immediately after the 20 min HIIT (*p* < 0.001) and 30 min HIIT (*p* = 0.001). Although BLa level was significantly decreased at 30 min post-HIIT compared with that measured at post-HIIT (*p* < 0.001), it was still significantly higher than baseline (*p* < 0.001). In addition, 30 min HIIT produced considerably higher BLa level than 20 min HIIT at both post-HIIT (*p* < 0.001) and 30 min post-HIIT (*p* = 0.005).

**TABLE 2:** Hematology and cognitive function values for subjects in the study (M ± SD).

	20 minutes HIIT			30 minutes HIIT			Time	Group	Group × Time
	Pre-HIIT	Post-HIIT	30 minutes Post-HIIT	Pre-HIIT	Post-HIIT	30 minutes Post-HIIT	F(p-value)[η^2^]	F(p-value)[η2]	F(p-value)[η^2^]
BLa(mmol/L)	0.90 ± 0.36	4.10 ± 1.82**^^	1.63 ± 0.43^**^##^^^^	0.86 ± 0.19	11.54 ± 5.48^**^	3.15 ± 1.38^**##^	32.080(<0.001) [0.865]	39.997(<0.001) [0.784]	18.258(<0.001)[0.785]
BDNF(ng/ml)	21.29 ± 1.17	26.66 ± 1.32^**^^^	21.27 ± 2.26^##^	21.83 ± 1.35	21.70 ± 1.54	20.86 ± 1.68^*#^	68.826(<0.001)[0.862]	9.082(0.012)[0.452]	43.159 (<0.001)[0.797]
VEGF-A(pg/ml)	549.44 ± 110.56	611.28 ± 107.28^**^	493.29 ± 154.67^#^^	550.82 ± 85.81	622.37 ± 83.43^**^	367.01 ± 91.84^**##^	217.955(<0.001)[0.978]	1.765(0.211)[0.138]	3.549(0.068)[0.415]
T(ng/ml)	8.93 ± 2.44	10.74 ± 3.09^*^	8.67 ± 2.73^##^^	8.23 ± 2.64	9.56 ± 3.64^*^	5.92 ± 1.51^**##^	17.780(0.001)[0.781]	2.563(0.138)[0.189]	2.482(0.133)[0.332]
C(ng/ml)	213.14 ± 37.77	254.26 ± 64.20^*^	207.05 ± 53.76^##^^	217.57 ± 46.73	317.06 ± 95.27^**^	297.97 ± 78.39^**^	18.955(<0.001)[0.791]	3.702(0.081)[0.252]	9.762(0.004)[0.661]
T/C	0.042 ± 0.011	0.043 ± 0.011	0.044 ± 0.014^^^^	0.038 ± 0.013	0.032 ± 0.015	0.021 ± 0.006*^*#^	8.860(0.002)[0.446]	7.346(0.020)[0.400]	9.002(0.001)[0.450]
RPE	6.833 ± 0.835	14.417 ± 1.165^**^^^	6.417 ± 0.515^##^	6.667 ± 0.651	16.417 ± 0.996^**^	6.500 ± 0.522^##^	621.792(<0.001)[0.985]	25.634(<0.001)[0.700]	18.293(<0.001)[0.624]
DST-F	8.25 ± 1.06	10.25 ± 1.144^**^^	10.17 ± 1.19^**^	8.92 ± 1.24	9.00 ± 1.04	9.17 ± 1.03	10.599(0.003)[0.679]	1.715(0.217)[0.135]	11.585(0.002)[0.699]
DST-B	6.42 ± 1.24	7.33 ± 1.37^*^	7.08 ± 1.08	5.83 ± 1.53	6.50 ± 1.93	6.58 ± 1.31	5.377(0.013)[0.328]	5.711(0.036)[0.342]	0.2339(0.790)[0.021]
Congruent task									
RT(ms)	1071.10 ± 166.90	855.04 ± 232.34**	841.46 ± 201.72^**^	973.44 ± 251.78	928.05 ± 282.12	984.21 ± 211.48	4.006(0.033)[0.267]	0.866(0.372)[0.073]	3.094(0.065)[0.220]
RA(%)	96.90 ± 3.96	98.44 ± 3.88	97.42 ± 4.86	96.86 ± 4.05	95.85 ± 6.86	95.83 ± 5.37	0.078(0.925)[0.015]	2.014(0.184)[0.155]	0.771(0.488)[0.134]
Neutral task									
RT(ms)	1046.40 ± 205.85	766.10 ± 158.67^**^^^	745.01 ± 104.30^**^^^	1003.49 ± 113.56	977.92 ± 155.88	1081.90 ± 238.47	15.946(0.001)[0.761]	8.910(0.012)[0.448]	18.026(<0.001)[0.783]
RA(%)	99.36 ± 2.22	97.81 ± 3.25	97.32 ± 4.12	98.31 ± 3.08	98.16 ± 4.75	98.20 ± 3.28	1.161(0.332)[0.095]	0.008(0.930)[0.001]	0.302(0.743)[0.027]
Incongruent task									
RT(ms)	1152.08 ± 177.27	958.09 ± 204.45^*^	1052.51 ± 229.63	1071.19 ± 322.49	1016.68 ± 257.68	1073.83 ± 267.60	2.018(0.157)[0.155]	<0.001(0.996)[<0.001]	0.498(0.615)[0.043]
RA(%)	94.36 ± 6.26	96.35 ± 4.77^^^^	94.18 ± 6.61^^^	91.52 ± 4.79	86.85 ± 3.92^*^	87.17 ± 4.34	1.242(0.308)[0.101]	30.323(<0.001)[0.734]	2.183(0.137)[0.166]

BLa, Blood lactate; BDNF, Brain-Derived neurotrophic factor; VEGF-A, Vascular endothelial growth factor-A; T, Testosterone; C, Cortisol; RPE, Rating of Perceived Exertion; DST-F, Digital Span test-forward; DST-B, Digital Span test-backward; RT, Response time; RA, Response accuracy.

*p < 0.05; **p < 0.01 vs. pre-HIIT; ^#^ *p* < 0.05, ^##^ *p* < 0.01 vs. post-HIIT; ^^^ *p* < 0.05, ^^*p* < 0.01vs. 30 minutes HIIT intervention.

### Serum BDNF and VEGF-A Responses to HIIT

As shown in Table 2, significant group by time of measurement interaction (F = 43.159, *p <* 0.001, η^2^ = 0.797), time (F = 68.826, *p <* 0.001, η^2^ = 0.862) and group (F = 9.082, *p* = 0.012, η^2^ = 0.452) main effects were found in BDNF analysis. The BDNF level was significantly elevated immediately after 20 min HIIT (*p* < 0.001) and returned to the baseline 30 min later. In contrast, 30 min HIIT did not immediately affect the BDNF level, but the BDNF level was significantly decreased after 30 min recovery (*p* = 0.045) and was even lower than its baseline (*p* = 0.013). Moreover, 20 min HIIT produced significant higher BDNF level than the 30 min HIIT at post-HIIT (*p* < 0.001).

As shown in Table 2, a significant time (F = 217.955, *p* < 0.001, η^2^ = 0.978) main effect was found in the analysis of VEGF-A. Compared with baseline, the VEGF-A level was significantly elevated immediately after both 20 min HIIT (*p* < 0.001) and 30 min HIIT (*p* < 0.001), and it returned to the baseline 30 min after 20 min HIIT. Whereas, the VEGF-A was significant decreased after 30 min recovery (*p* < 0.001) and was even lower than the baseline (*p* < 0.001) in 30 min HIIT group. Furthermore, 20 min HIIT induced considerably higher level of VEGF-A than 30 min HIIT at 30 min post-HIIT (*p* = 0.014).

### Testosterone, Cortisol, T/C and RPE Responses to HIIT

As shown in Table 2, a significant time (F = 17.780, *p* = 0.001, η^2^ = 0.781) main effect was found in the analysis of serum testosterone. The level of testosterone was significantly increased immediately after both 20 min HIIT (*p* = 0.012) and 30 min HIIT (*p* = 0.045) compared with their baselines. The 20 min HIIT induced increased testosterone level was declined back to the baseline after 30 min recovery, while the 30 min HIIT induced increased testosterone level was significantly decreased after 30 min recovery (*p* = 0.003) and was even lower than the baseline (*p* = 0.006). Group comparison indicated that the serum testosterone level measured 30 min after training was considerably higher in 20 min HIIT group than in 30 min HIIT group (*p* = 0.012).

As shown in Table 2, significant group by time of measurement interaction (F = 9.762, *p* = 0.004, η^2^ = 0.661) and time (F = 18.955, *p* < 0.001, η^2^ = 0.791) main effects were found in serum cortisol. Both 20 min HIIT (*p* = 0.044) and 30 min HIIT (*p* = 0.006) significantly upregulated the serum cortisol level immediately. After 30 min recovery, the serum cortisol returned to baseline in the 20 min HIIT group, while the serum cortisol maintained increased level in the 30 min HIIT group. What is more, the serum cortisol concentration was considerably lower at 30 min post-HIIT in the 20 min HIIT group compared with that in the 30 min HIIT group (*p* = 0.012).

As indicated in Table 2, significant group by time of measurement interaction (F = 9.002, *p* = 0.001, η^2^ = 0.450), time (F = 8.860, *p* = 0.002, η^2^ = 0.446) and group (F = 7.346, *p* = 0.020, η^2^ = 0.400) main effects were found in the testosterone/cortisol ratio (T/C). There were no significant differences in T/C among three time points in 20 min HIIT group. In 30 min HIIT group, the T/C did not change immediately after training but decreased significantly after 30 min recovery (*p* = 0.020) and was even lower than the baseline (*p* < 0.001). Group comparison indicated that the T/C in 20 min HIIT group was considerably higher than 30 min HIIT group at 30 min post-HIIT (*p* < 0.001).

### CWST Responses to HIIT

As seen in Table 2, significant time (F = 4.006, *p* = 0.033, η^2^ = 0.267) main effects were found in the RT of congruent tasks. RT for congruent tasks was significantly shorter immediately after the 20 min HIIT than pre-HIIT (*p* < 0.001), this shortened RT was maintained for 30 min following 20 min HIIT (*p* < 0.001). Significant group by time of measurement interaction (F = 18.062, *p* < 0.001, η^2^ = 0.783), time (F = 15.946, *p* = 0.001, η^2^ = 0.761) and group (F = 8.910, *p* = 0.012, η^2^ = 0.448) main effects were found in the RT of neutral tasks. RT for neutral tasks was significantly shorter immediately after the 20 min HIIT than pre-HIIT (*p* < 0.001), and this shortened RT was also maintained for 30 min following 20 min HIIT (*p* < 0.001). Moreover, 20 min HIIT contributed to significant shorter RT at post-HIIT (*p* = 0.005) and 30 min post-HIIT (*p* = 0.001) than 30 min HIIT. No significant main effect was found in the RT of incongruent tasks. RT for incongruent tasks was significantly shorter immediately after the 20 min HIIT than pre-HIIT (*p* = 0.043).

Significant group by time of measurement interaction (F = 30.323, *p* < 0.001, η^2^ = 0.734) effects were found in the RA of incongruent tasks. The RA of congruent and neutral tasks did not differ significantly between the 20 and 30 min HIIT protocols throughout the experimental sessions. The RA was significantly decreased only for incongruent task immediately after 30 min HIIT when compared to pre-HIIT (*p* = 0.035). Importantly, 30 min HIIT resulted in significant lower RA at post-HIIT (*p* < 0.001) and 30 min post-HIIT (*p* = 0.014) than 20 min HIIT.

### DST Responses to HIIT

As seen in Table 2, significant group by time of measurement interaction (F = 11.585, *p* = 0.002, η^2^ = 0.699) and time (F = 10.599, *p* = 0.003, η^2^ = 0.679) main effects were found in the DST-F. The participants got significant higher averaged score right after 20 min HIIT (*p* < 0.001) and also got considerably higher score 30 min later (*p* = 0.002), compared with baseline. In contrast, there were no significant differences in DST-F scores among three time points in 30 min HIIT group. Group comparison indicated that the DST-F score was considerably higher right after the 20 min HIIT, compared with the 30 min HIIT intervention (*p* = 0.024).

As shown in Table 2, significant group (F = 5.711, *p* = 0.036, η^2^ = 0.342) and time (F = 5.377, *p* = 0.013, η^2^ = 0.328) main effects were found in the DST-B. The DST-B averaged score was significantly elevated only after the 20 min HIIT (*p* = 0.026) and returned to baseline 30 min later. Whereas, it was not changed after 30 min HIIT.

### Relationship Between Changes From Baseline Variables (ΔBDNF, ΔVEGF-A and ΔBLa)

**FIGURE 3:**
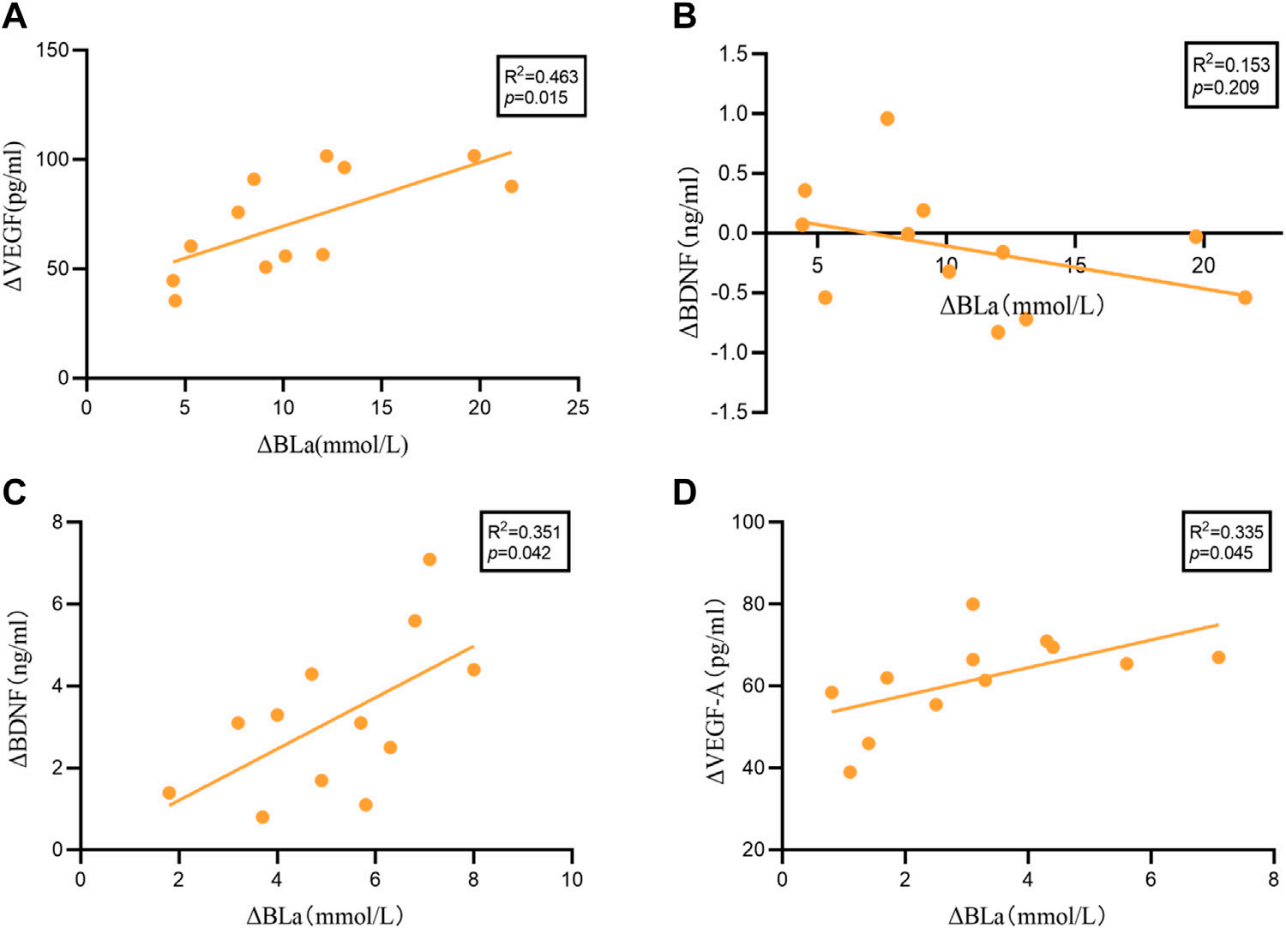
The results of the linear regression analysis. **(A)**: ΔBLa and ΔVEGF-A in 30 minutes HIIT intervention. **(B)**: ΔBLa and ΔBDNF in 30 minutes HIIT intervention. **(C)**: ΔBLa and ΔVEGF-A in 20 minutes HIIT intervention. **(D)**: ΔBLa and ΔBDNF in 20 minutes HIIT intervention.

We performed linear regression analysis to examine the association between ΔBDNF (20 min HIIT = 5.17 ± 1.79; 30 min HIIT = -0.13 ± 0.51), ΔVEGF-A (20 min HIIT = 61.83 ± 11.11; 30 min HIIT = 71.56 ± 23.66) and ΔBLa (20 min HIIT = 3.20 ± 1.90; 30 min HIIT = 10.68 ± 5.50), (*Δ* = post-HIIT-pre-HIIT). As shown in Figure 3 significant relationship was identified between ΔBLa and ΔVEGF-A (*R* ^2^ = 0.463, *p* = 0.015), while no significant relationship was found between ΔBLa and ΔBDNF (*R* ^2^ = 0.153, *p* = 0.209) in 30 min HIIT group. ΔBLa was statistically related to ΔBDNF (*R* ^2^ = 0.351, *p* = 0.042) and ΔVEGF-A (*R* ^2^ = 0.335, *p* = 0.045) in 20 min HIIT group.

## Discussion

The main finding in the present study is that the duration of HIIT affects both neurotrophins and cognitive function. In other words 20 min HIIT leads to more beneficial response of cognitive function as well as serum BDNF and VEGF-A levels than 30 min HIIT in healthy young men.

As a protein member of the neurotrophin family, BDNF is derived mainly from the brain, however, its receptors are also found in a wide variety of peripheral tissues such as liver, pancreas, adipose tissue, heart, endocrine system, reproductive system, smooth muscle and skeletal muscle (Hallböök et al., 1991; Scarisbrick et al., 1993; Bathina et al., 2016; Matsumoto et al., 2021). Previous studies have indicated that BDNF passes through the blood-brain barrier in a bidirectional transport manner (Rasmussen et al., 2009). Therefore, alterations in periphery BDNF levels could reflect the variation of brain BDNF (Klein et al., 2011). Previous studies have shown that BDNF is sensitive to exercise (Cabral-Santos et al., 2016). In the present study, 20 min HIIT intervention elevated BDNF level in the serum at post-HIIT and the BDNF level was returned to the baseline at 30 min post-HIIT. However, as the effect of 30 min HIIT intervention, there was no significant difference in the serum BDNF concentration at post-HIIT, but it was decreased significantly at 30 min post-HIIT. Moreover, we found a positive correlation between ΔBLa and ΔBDNF in 20 min HIIT group, but there was no correlation between ΔBLa and ΔBDNF in 30 min HIIT group. García-Suárez et al. (2020) have reported similar findings that serum cortisol and BLa of healthy women increased immediately after a bout of 12 min HIIT, while serum BDNF levels did not change following HIIT. Moreover, Rojas Vega et al. (2006) found that serum BDNF of healthy male athletes elevated immediately following a bout of ramp incremental cycle ergometry, but serum cortisol levels did not change following HIIT.

It has been pointed out that BLa is a key factor inducing BDNF synthesis (El Hayek et al., 2019; Müller et al., 2020), however, cortisol is a key factor suppressing BDNF synthesis (Issa et al., 2010; Kino et al., 2010; García-Suárez et al., 2020). Therefore, we speculate that BLa and serum cortisol might contribute to the different effects of HIIT with different duration on BDNF. In the present study, we found that the increase of concentrations of BLa and serum cortisol occurred in a duration-dependent manner, as higher BLa and cortisol levels were produced by a longer-duration training in the same intensity. Thirty minutes HIIT maintained temporal differences that is induced higher BLa and cortisol levels.

Moreover, testosterone has been shown to have a wide range of neuroprotective effect by increasing levels of BDNF and VEGF within the brain (Spritzer and Roy, 2020). Both interventions significantly evaluated testosterone level at post-HIIT, but after 30 min recovery, 20 min HIIT maintained higher level of testosterone than 30 min HIIT. Since testosterone shows anabolic effects and cortisol promotes catabolic effects, the T/C has been considered as a signal of the training activity is too high and catabolic processes prevail (Ambroży et al., 2021). T/C is correlated with the duration and intensity of training, T/C decline indicates that the body is under high metabolic stress and accumulates fatigue (Turner et al., 2017). In this study the ratio of T/C declined gradually over time in the 30 min HIIT group, while there was no significant change in the 20 min HIIT group, and 30 min HIIT produced higher RPE than 20 min HIIT (20 min HIIT: 14.417 ± 1.165; 30 min HIIT: 16.417 ± 0.996) indicating that the body is under higher physiological strain from training in 30 min HIIT than 20 min HIIT.

It has been reported that high intensity exercise may provoke an increase in circulating cortisol level and therefore increase arousal, which might lead to impaired cognitive performance (Hill et al., 2008; Quintero et al., 2018). Thus, the inhibitory effect on the synthesis of BDNF induced by cortisol may be greater than the stimulative effect induced by BLa in 30 min HIIT group. On the contrary, the stimulative effect induced by BLa on the synthesis of BDNF may be greater than the inhibitory effect induced by serum cortisol in the 20 min HIIT group. Given the antagonistic effects on BDNF synthesis produced by exercise-induced BLa and serum cortisol, exercise-induced BLa and serum cortisol must maintain optimal levels to produce BDNF for improving cognitive function.

In the present study, both 20 min HIIT and 30 min HIIT elevated serum VEGF-A immediately following HIIT. The serum VEGF-A returned to baseline 30 min after HIIT in the 20 min HIIT group, while the serum VEGF-A significantly decreased in the 30 min HIIT group. Hebisz et al. (2019) reported a similar finding that the serum VEGF-A of cyclists was downregulated after a bout of sprint interval training (4 × 30s all-out repetitions interspersed with 90 s of rest). It has been reported that the serum VEGF-A plays a role in tissue repair (Tischer et al., 1989). It can be surmised that the body is under high metabolic stress and this downregulation is because of local tissue injury 30 min after HIIT. Moreover, the decreases of serum BDNF and VEGF-A after HIIT indicate their utilization for local tissue repair (Nofuji et al., 2008; Hebisz et al., 2019).

The current study indicated that HIIT-induced BLa was positively correlated with the increased peripheral level of VEGF-A in both HIIT groups. Previous literature has demonstrated that serum VEGF-A is highly correlated with BLa. Kujach et al. (2019) observed a significant elevation across time in VEGF-A and BLa in young males after 6 × 30s sprint interval training, and BLa was positively correlated with increased peripheral level of VEGF-A. Morland et al. (2017) showed that l-lactate subcutaneous injection and HIIT leading to the increases in BLa levels, increases brain VEGF-A expression and capillary density in wild-type mice, but not in knockout mice lacking HCAR1 (a receptor of lactate).

Recently, a meta-analysis suggests that participation in HIIT can improve adolescents’ cognitive function and mental health. Previous studies have suggested a dose-response relation between exercise intensity and cognitive performance after exercise (Kamijo et al., 2004; Chang and Etnier, 2009; Chang et al., 2011). Our results showed that there was also a dose-response relation between exercise duration and cognitive performance after exercise, that is, 20 min HIIT stimulated executive function, improving CWST performance by a short response time and elevating DST score, but 30 min HIIT could not. Importantly, the findings of this study suggest that exercise duration of 20 min, with 5-min warm-up and cool-down as recommended by the ACSM, results in the largest benefits to cognitive function. On the other hand the facilitative effect of 20 min acute moderate-intensity exercise on cognitive performance partly attributes to the expression of neurotrophins, and acute exercise induces greater attentional allocation, more efficient information processing speed, and optimal physiological and psychological arousal occurs (Tsai et al., 2014; Hwang et al., 2016; Bettio et al., 2019; Chen et al., 2019; Tian et al., 2021). We observed that the RA of incongruent tasks performed immediately after 30 min HIIT was significantly reduced compared to that at pre-HIIT. Moreover the incongruent condition resulted in longer RT and less RA than the congruent condition in CWST. This finding is consist with a previous study, Tsukamoto et al. found that RA was significantly decreased for incongruent task immediately after 28 min HIIT (4 × 4 min training interspersed with 3 min of active recovery) (Tsukamoto et al., 2016), suggesting that prolonged-intense exercise (30 min HIIT) might leads to damage on higher-order aspects of cognition in general. Hwang er al. (2016) found that shorter response times in Trail Making Test Part-B were significantly correlated with an increase in ΔBDNF after 20 min high-intensity exercise in healthy adults. Unfortunately, our study did not show the cognitive function was correlated with BDNF and VEGF-A. The heterogeneous results might be due to the differences in methodologies involving exercise protocol, cognitive task type, test time points, participant’s cardiorespiratory fitness level and other confounding factors.

### Limitations

There are some limitations to be considered: First, CWST and DST are adopted to evaluate inhibition and updating of executive function respectively in this study. Whereas, executive function is a comprehensive cognitive area that consists of several subcomponents of cognitive performance, namely inhibition, updating, and shifting. Although a meta-analysis suggest that acute HIIT generally tends to have not positive effect on shifting (Ai et al., 2021), future studies are necessary to explore how effects are similar or different depending on the particular type of cognitive task. Second, the potential neurobiological mechanisms of acute exercise promoting cognitive function include exercise-induced neurotrophins and increases in general physiological arousal or neural activation (Hwang et al., 2016). This study focuses on neurotrophins, HIIT may lead to activation of different brain regions, so further studies will use neuroelectric techniques to explore the relationship between neurotrophins and regional neural activity. Furthermore, our subjects are right-handed young man, more heterogeneous subjects in a larger sample size will be necessary to be recruited in further study to assess the broad applicability of our findings.

## Conclusion

Twenty minutes HIIT is more effective than 30 minutes HIIT for promoting serum levels of BDNF and VEGF-A, and cognitive function in healthy young men. In addition, both serum ΔBDNF and ΔVEGF-A in 20 min HIIT group were positively associated with ΔBLa, while only serum ΔVEGF-A was positively associated with ΔBLa in the 30 min HIIT group.

## Data Availability

The raw data supporting the conclusions of this article will be made available by the authors, without undue reservation.
